# Adsorption of Chromate Ions by Layered Double Hydroxide–Bentonite Nanocomposite for Groundwater Remediation

**DOI:** 10.3390/nano12081384

**Published:** 2022-04-18

**Authors:** Yoogyeong Kim, Yeongkyun Son, Sungjun Bae, Tae-Hyun Kim, Yuhoon Hwang

**Affiliations:** 1 Department of Environmental Engineering, Seoul National University of Science and Technology, Seoul 01811, Korea; keo0610@seoultech.ac.kr (Y.K.); clayton7167@naver.com (Y.S.); 2 Department of Civil and Environmental Engineering, Konkuk University, Seoul 05029, Korea; bsj1003@konkuk.ac.kr

**Keywords:** layered double hydroxide, bentonite, nanocomposite, chromate adsorption, groundwater remediation

## Abstract

Herein, magnesium/aluminum-layered double hydroxide (MgAl-LDH) and bentonite (BT) nanocomposites (LDH–BT) were prepared by co-precipitation (CP), exfoliation–reassembly (ER), and simple solid-phase hybridization (SP). The prepared LDH–BT nanocomposites were preliminarily characterized by using powder X-ray diffractometry, scanning electron microscopy, and zeta-potentiometry. The chromate adsorption efficacies of the pristine materials (LDH and bentonite) and the as-prepared nanocomposites were investigated. Among the composites, the LDH–BT_SP was found to exhibit the highest chromate removal efficiency of 65.7%. The effect of varying the LDH amount in the LDH–BT composite was further investigated, and a positive relationship between the LDH ratio and chromate removal efficiency was identified. The chromate adsorption by the LDH–BT_SP was performed under various concentrations (isotherm) and contact times (kinetic). The results of the isotherm experiments were well fitted with the Langmuir and Freundlich isotherm model and demonstrate multilayer chromate adsorption by the heterogeneous LDH–BT_SP, with a homogenous distribution of LDH nanoparticles. The mobility of the as-prepared LDH–BT_SP was investigated on a silica sand-filled column to demonstrate that the mobility of the bentonite is dramatically decreased after hybridization with LDH. Furthermore, when the LDH–BT_SP was injected into a box container filled with silica sand to simulate subsurface soil conditions, the chromate removal efficacy was around 43% in 170 min. Thus, it was confirmed that the LDH–BT prepared by solid-phase hybridization is a practical clay-based nanocomposite for in situ soil and groundwater remediation.

## 1. Introduction

Chromium (Cr) has been used in various industries, such as electroplating, dyeing, cement production, mining, photography, etc. [1,2]. However, the presence of chromium in ecosystems or environments has triggered severe problems for animals and human beings [3,4]. For example, the high concentration of chromium in the ecosystem inhibits the growth of plants by preventing the absorption of nutrients [5]. Moreover, chromium species can cause serious diseases, such as lung cancer, nasal irritation, nasal ulcers, hypersensitivity reactions, and contact dermatitis [6,7]. Chromium has been shown to exist in aqueous systems in the form of oxyanions such as hydrogen chromate (HCrO_4_^−^), chromate (CrO_4_^2−^), and dichromate (Cr_2_O_7_^2−^), depending on the pH of the solution [8]. In particular, the concentration of hexavalent chromium in drinking water was regulated by the World Health Organization (WHO) as a maximum of 0.05 mg/L [9]. Nevertheless, high concentrations of hexavalent chromium were widely founded in industrial wastewater and groundwater wells around the world [10,11].

The removal of hexavalent chromium from wastewater, surface water, and groundwater has attracted the attention of many researchers [12,13,14], and various techniques, such as precipitation [15], ion exchange [16], reverse osmosis [17], reduction [18], and adsorption [19], have been employed. While most of these techniques incur high treatment costs and are unsuitable for large-scale industrial or water treatment plants, adsorption is attractive and widely used, due to its high efficiency, convenience, and cost-effectiveness [20,21,22]. Many researchers have investigated various adsorbents, such as zeolites, carbon-based materials, clays, and layered double hydroxides, for effective chromate ion adsorption [23,24,25,26]. In particular, clay materials such as bentonite have been intensively studied, due to their characteristic properties, such as cation exchange capacity, low cost, and eco-friendliness [27,28]. However, the adsorption of chromate ions onto bentonite is limited by its negative surface charge in the aqueous system. To address this problem, surface modification or hybridization with positively charged organic/inorganic moieties is necessary [29,30,31]. However, despite enhancing the chromate adsorption efficacy, the immobilized organic moieties could be released during the adsorption process, thereby leading to another environmental issue.

The anionic clays known as layered double hydroxide (LDH) have been extensively studied, due to their high surface area, basicity, anion exchange properties, and positive surface charge [32,33]. An LDH is a hydrotalcite-like compound with the general formula [M^II^_1−x_M^III^_x_(OH)_2_]^x+^[A^n−^]_x/n_·yH_2_O, where M^II^ and M^III^ are divalent and trivalent metal cations, respectively, and A^n−^ is an anion. Due to the advantages of the LDH, many researchers have studied their adsorption of chromate ions under various conditions (pH, chromate concentration, large dosing amount, etc.) [34,35,36,37]. In spite of those advantages, nanomaterials, including LDH, might have potential disadvantages, such as stability of materials followed unexpected toxicity to eco-systems, attributed to by their tens to hundreds of nm size [38,39]. To prevent the possible side effect and enhance characteristic properties of not only LDH but also nanomaterials, there are some reports on the hybridization of LDH with various support materials, such as magnetite [40], biochar [41], and graphene [42], for environmental remediation. There are few studies on the bentonite (BT), natural clay, and LDH hybrid materials to enhance the adsorption properties in aqueous systems, such as heavy metal ions and dye molecules for environmental remediation [43,44]. These have mostly prepared the LDH–BT nanocomposite via the conventional co-precipitation method. Although this is a facile method, it does not allow for easy control of the metal ratio and the size or morphology of the LDH during the reaction. An alternative eco-friendly and cost-effective hybridization methodology might be that of solid-phase hybridization. For example, Kim et al. reported the successful intercalation of 2-aminoethanesulfonate, known as taurine, into the interlayer space of a Ca^2+^-containing LDH [45] through solid-phase intercalation.

In the present study, LDH–BT nanocomposites were prepared via three different methodologies, namely (i) co-precipitation, (ii) exfoliation–reassembly, and (iii) solid-phase hybridization, and their chromate adsorption efficacies were evaluated. The as-prepared LDH–BT nanocomposites were preliminarily characterized by powder X-ray diffractometry (PXRD), scanning electron microscopy (SEM), and zeta-potentiometry. To investigate the chromate adsorption efficacy, chromate adsorption experiments were performed with various concentrations of chromate (2–100 mg/L) in aqueous solution (isotherm) and for various contact times (0.5–240 min; kinetic). The obtained isotherm results were analyzed by using the Langmuir and Freundlich isotherm models. Moreover, the mobility and chromate adsorption efficacy were evaluated under simulated subsurface conditions. To the best of the present authors’ knowledge, this is the first study to evaluate the practical applicability of LDH–BT prepared via solid-phase intercalation in the simulated soil box-test for in situ groundwater remediation.

## 2. Experimental

### 2.1. Materials

Magnesium chloride hexahydrate (MgCl_2_·6H_2_O, 99%), aluminum chloride hexahydrate (AlCl_3_·6H_2_O, 98%), and potassium chromate (K_2_CrO_4_, 98.5%) were obtained from Samchun Chemicals Co., Ltd. (Seoul, Korea). Sodium chloride (NaCl, 99%) and sodium hydroxide (NaOH, 99%) were purchased from Duksan Chemical (Ansan, Korea). Bentonite, 1,5-diphenylcarbazide (C_13_H_14_N_4_O), acetone, and sulfuric acid (H_2_SO_4_, 95–98%) were purchased from Sigma-Aldrich Co. LLC (St. Louis, MO, USA). All the chemicals were used without further purification. Ultrapure deionized (DI) water was produced by using a water purification system (Synergy^®^, Merck, Kenilworth, NJ, USA).

### 2.2. Synthesis of MgAl-Cl Layered Double Hydroxide

For the preparation of the MgAl-Cl LDH (denoted hereafter as LDH), 200 mL of mixed metal solution (24.4 g of MgCl_2_·6H_2_O and 14.5 g of AlCl_3_·6H_2_O) was prepared and placed in a 500 mL round-bottomed flask. A 250 mL alkaline solution (20.0 g of NaOH and 10.5 g of NaCl) was then prepared and added drop-wise into the mixed metal solution with vigorously stirred until a pH of around 9.5 was achieved. This slurry was then aged at room temperature for 24 h, under a N_2_ atmosphere. Subsequently, the obtained product was collected by centrifugation, washed three times, and lyophilized. All preparations and procedures were performed by using decarbonated water prepared by bubbling N_2_ gas through boiling water for over 30 min.

### 2.3. Preparation of LDH–BT Nanocomposites

#### 2.3.1. LDH–BT by Co-Precipitation (LDH–BT_CP)

For co-precipitation, the bentonite (300 mg) was dispersed in DI water (300 mL) for 24 h, collected by centrifugation, and re-dispersed in decarbonated water (100 mL) in a 250 mL round-bottomed flask. Based on the calculation for 150 mg of LDH for hybridization, 2.44 g of MgCl_2_·6H_2_O and 1.45 g of AlCl_3_·6H_2_O were added to the bentonite slurry. Then 200 mL of alkaline solution (2.00 g of NaOH and 1.05 g of NaCl) was added drop-wise to the bentonite and mixed metal slurry until the pH was around 9.5. The obtained slurry was then aged at room temperature for 24 h, under a N_2_ atmosphere. Finally, the LDH–BT_CP was collected by centrifugation, washed two times, and dried with a lyophilizer.

#### 2.3.2. LDH–BT by Exfoliation–Reassembly (LDH–BT_ER)

First, the prepared LDH (300 mg) was dispersed in formamide solution (300 mL), with vigorous stirring, under a N_2_ atmosphere, for 24 h, for exfoliation of the LDH sheets. Next, the bentonite (600 mg) was dispersed in decarbonated water (600 mL) and stirred for 24 h. The exfoliated LDH suspension was then collected by centrifugation and re-dispersed with bentonite swelling solution. Finally, the reaction vessel was stirred vigorously under a N_2_ atmosphere, for 24 h, at room temperature. The LDH–BT_ER was collected by centrifugation, washed two times, and then dried with a lyophilizer.

#### 2.3.3. LDH–BT by Solid-Phase Hybridization (LDH–BT_SP)

For solid-phase hybridization, the powdered bentonite (600 mg) and as-prepared LDH (300 mg, 50 wt.%) were homogenously ground in an agate mortar for 5 min. To optimize the reaction conditions of the LDH–BT_SP, the weight percent of LDH with respect to bentonite was varied.

### 2.4. Characterization

The PXRD patterns were obtained by using a Bruker DE/D8 Advance (Bruker A.X.S. GmbH, Berlin, Germany) with Cu K_α_ radiation (λ = 1.5418 Å). The diffraction patterns were collected in the range of 3° to 80°, with a 5 mm air-scattering slit, a 2.6 mm equatorial slit, and increments of 3.9°/min. The d-spacings of the prepared samples were calculated by Bragg’s equation (Equation (1)):

*nλ = 2d·sinθ* (1)

where *n* = order of reflection, *λ* = wavelength of radiation, *d* = interlayer space, and *θ* = Bragg’s diffraction angle.

The metal (Mg^2+^ and Al^3+^) ratios in the pristine LDH, the bentonite, and the three nanocomposites were analyzed by inductively coupled plasma–optical emission spectroscopy (ICP–OES; 5110 SVDV, Agilent Technologies, Inc., Santa Clara, CA, USA). For ICP–OES, around 20 mg of the samples were digested with mixed acid solution (HNO_3_ and HCl) and then treated in a microwave reactor (Multiwave 7000, Anton Paar GmbH, Graz, Austria) at 180 °C for 40 min. The Mg^2+^ and Al^3+^ ratios in nanocomposites were then obtained by calculation, excluding the Mg^2+^ and Al^3+^ from the parent bentonite. The zeta-potentials of the various samples were measured via electrophoretic light scattering with an ELS-Z2000 (Otsuka Electronics, Osaka, Japan). For the measurement, powdered samples were dispersed in DI water (~0.1 mg/mL) and sonicated for 15 min. The average zeta-potential value was calculated by the program provided by Otsuka electronics, using the Smoluchowski equation. The morphologies of the bentonite; the LDH; and the LDH–BT_CP, _ER, and _SP samples were investigated via high-resolution field-emission scanning electron microscopy (HR-SEM), using a Hitachi SU8010 (Hitachi High-Technologies Corporation, Tokyo, Japan), with a 15 kV accelerated electron beam at a working distance of 8 mm.

### 2.5. Chromate Adsorption Experiments

First, the chromate adsorption efficacies of the various as-prepared composite samples were compared, and the LDH–BT_SP with 50% LDH by weight of bentonite was identified as the optimum composite. In this screening procedure, the initial chromate concentration and adsorbent dosage were set to 10 mg/L and 1.0 g/L, respectively. The sample was withdrawn after 2 h of adsorption time, and the collected suspension was filtrated with a syringe filter (PES, 0.45 μm). The concentration of chromate in the supernatant was quantified by the diphenylcarbazide method, using UV–Vis spectroscopy (Genesys50, Thermo, Waltham, MA, USA), at a wavelength of 540 nm [10].

The chromate adsorption kinetics of the LDH–BT_SP with 50 wt.% LDH were then studied by evaluating the chromate adsorption efficiency according to contact time. For this procedure, 300 mg of LDH–BT_SP was dispersed into 300 mL of chromate solution placed in a 500 mL round-bottom flask. The initial chromate concentration was set as 10 mg/L, and the sample was collected at designed time points (0.5, 1, 3, 5, 15, 30, 60, 120 and 240 min). The chromate concentration was quantified by the diphenylcarbazide method after sample filtration.

To evaluate the chromate adsorption capacity of the LDH–BT_SP with 50 wt.% LDH according to chromate concentration, batch isotherm experiments were performed with various chromate concentrations (2, 5, 10, 20, 50, 75, and 100 mg/L). For this procedure, 30 mg of LDH–BT_SP was dispersed in chromate solution (30 mL) and vigorously shaken by a vertical shaker (35 rpm), at room temperature. After 2 h, the supernatant was collected by a syringe filter (PES, 0.45 μm) and quantified by the diphenylcarbazide method. The obtained isotherm result was fitted with the Langmuir isotherm [46] (Equation (2)) and the Freundlich isotherm [47] (Equation (3)) models:

*q_e_* = (*q_m_a_L_C_e_*)/(1 + *a_L_C_e_*) (2)

*q_e_* = *K_F_·C_e_^(1/n)^* (3)

where *q_e_* is the quantity of adsorbate adsorbed per unit weight of solid adsorbent, *q_m_* is the maximum adsorption capacity of the adsorbent (mg/g), *C_e_* is the equilibrium concentration of the adsorbate in solution (mg/L), *a_L_* is the Langmuir affinity constant (L/mg), and *K_F_* and *1*/*n* are constants indicating the adsorption capacity and the adsorption intensity, respectively.

In addition, the dimensionless separation factor (*R_L_*) was calculated by using Equation (4) [48]:

*R_L_* = 1/(1 + *a_L_C_0_*) (4)

where *C_0_* is the highest initial adsorbate concentration (mg/L), and *a_L_* is the Langmuir constant.

### 2.6. Mobility Test

The mobilities of the pristine bentonite and the LDH–BT_SP were evaluated under simulated soil conditions, using a column (ϕ 3 cm × 15 cm) filled with silica sand (<0.3 mm, Joomoonjin silica sand Co., Ltd., Gangneung, Korea). First, 550 mg of the powdered samples was dispersed into 550 mL of DI water and vigorously stirred with a magnetic stirrer for 1 h. Then, DI water was passed through the prepared column by using a peristatic pump (5.0 mL/min), until the silica sand was wet. Then the prepared suspension (1.0 g/L) was injected into the column via a peristatic pump (5.0 mL/min). Finally, samples of the solution were collected from the column outlet at 5 min intervals for 100 min. The mobilities were then calculated as the difference in absorbance between the initial sample suspension (I_0_) and the collected sample (I) at 700 nm via UV–Vis spectrophotometry.

### 2.7. Box Test

To evaluate the chromate adsorption under simulated soil conditions, a polyacrylate box (3 cm × 8 cm × 22 cm) was prepared with three inlet ports and one outlet port, as shown in Supplementary Figure S1. An injection port was provided at the top-center of the box for injecting the LDH–BT_SP suspension. The prepared box was entirely filled with silica sand. For the control experiment, the chromate solution (2.5 mg/L) was injected into the box by a peristaltic pump, at a flow rate of 9.0 mL/min for 170 min, and samples were collected every 10 min for a total of 170 min. At the end of this experiment, the box tester was flushed with DI water for 1 h to ensure that no chromate solution remained in the box. For the active experiment, around 1 g of the LDH–BT_SP was dispersed in DI water (100 mL) and stirred for over 30 min by a magnetic stirrer. The silica sand was wetted with DI water via the peristaltic pump (9.0 mL/min), and then the as-prepared LDH–BT_SP suspension was injected by using a syringe at the top-center of the box. After that, 1 L of chromate solution (2.5 mg/L) was added via a peristaltic pump (9.0 mL/min), and samples were collected every 10 min for a total of 170 min. The concentration of chromate in the samples was quantified by the diphenylcarbazide method.

## 3. Results and Discussion

### 3.1. Characterization of the Parent Bentonite, the LDH, and the Various LDH–BT Nanocomposites

**Figure 1:**
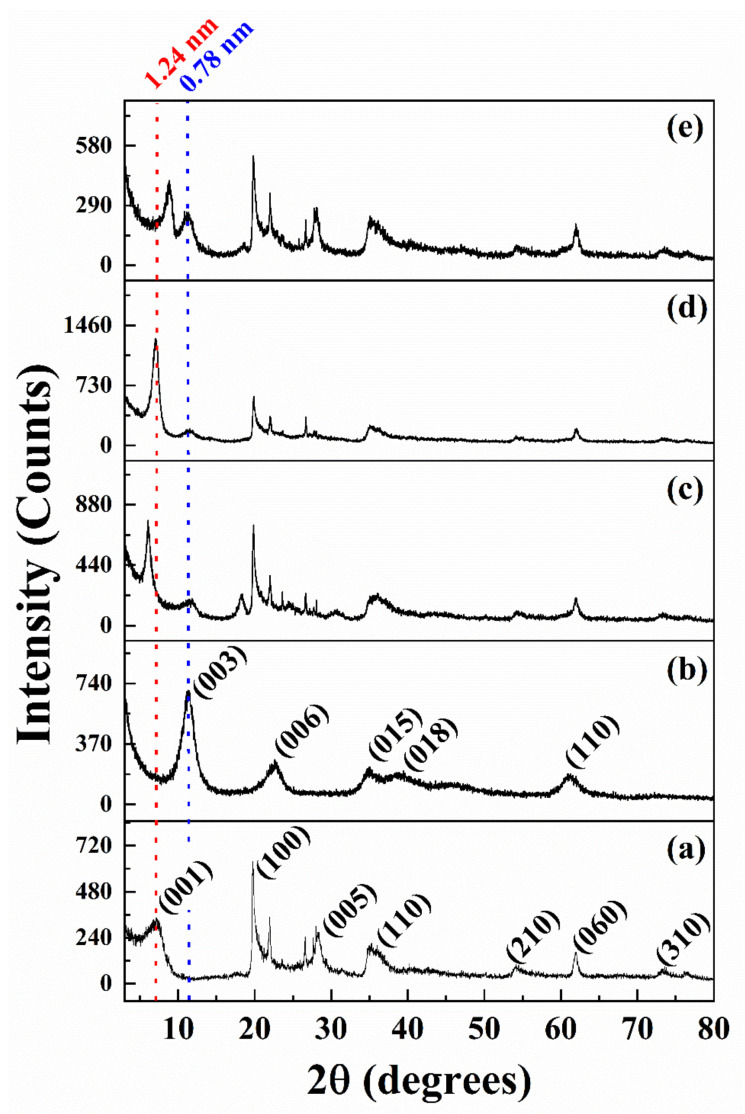
The PXRD patterns of (**a**) bentonite, (**b**) LDH, (**c**) LDH–BT_CP, (**d**) LDH–BT_ER, and (**e**) LDH–BT_SP (red dotted line: position of (001) from bentonite, blue dotted line: position of (003) from LDH).

The detailed crystal structures of the starting materials and the various composites were revealed by the PXRD results in Figure 1 and Table 1. Thus, in Figure 1a, the bentonite exhibits the typical diffraction pattern, with peaks at 7.46,19.72, 28.26, 35.07, 54.27, 61.95 and 76.44°, corresponding to the (001), (100), (005), (110), (210), (060), and (310) crystal planes, respectively (JCPDS No. 03-0019), as reported in previous studies [49,50]. In Figure 1b, the LDH also shows a well-developed (003) diffraction peak at 11.39°, which is attributed to the layer stacking order, along with other peaks, due to the (006), (015), (018), and (110) planes. After hybridization, the three nanocomposites exhibited two characteristic diffractions at around 6.1–8.9 and 11.4° that were attributed to the (001) plane of bentonite and the (003) plane of LDH, respectively. However, the (001) diffraction peak was shifted from 7.46° in the pristine bentonite to 6.09° in the LDH–BT_CP, indicating an expansion of the interlayer space from 1.24 to 1.45 nm, respectively (Table 1). This might be due to the partial incorporation of co-precipitated LDH nanoparticles between the bentonite layers [51]. The diffraction peaks of the LDH–BT_ER (Figure 1d) maintained the same positions as those of the parent bentonite and LDH, and the d-spacing did not change significantly. Meanwhile, the LDH–BT_SP showed a decrease in the interlayer space and a corresponding shift of the (001) diffraction peak to a higher angle (8.9°). This decrement of interlayer space was contributed to by the loss of water molecules from the bentonite interlayer, due to the solid phase hybridization with more hydrophilic LDH nanoparticles [52,53].

The intensity and full width at half maximum (FWHM) of the (00l) diffractions of layered materials generally indicate the crystallinity and *c*-axis stacking order. The results in Table 1 reveal that the FWHM values of the bentonite were dramatically decreased by 26–50% after hybridization with LDH, thereby indicating that the bentonite layers were well stacked in the composite materials. By contrast, the FWHM values of the (003) diffraction of LDH were unchanged, thereby indicating that the stacking order of LDH was not affected by hybridization with bentonite. Furthermore, the intensity ratio of the (001) peak to the (003) peak (i.e., BT/LDH) in the LDH–BT_SP is 1.75:1, and it is 2.7 and 5.8 times lower than that of the LDH–BT_CP and LDH–BT_ER, respectively. These results indicate that the solid-phase hybridization method is more effective than the co-precipitation and exfoliation–reassembly methods at preserving the crystallinity of each layered material.

The metal ratios of LDH in the LDH–BT_CP, _ER, and _SP samples after excluding the Mg^2+^ and Al^3+^ from the parent bentonite in the ICP-OES results are shown in Table 1. As summarized in Table 1, the parent LDH exhibits an Mg:Al ratio of 2.13:1, which matches the designed reaction conditions of 2:1. After hybridization, the Mg:Al ratios of the LDH–BT_CP, _ER, and _SP nanocomposites were determined as 1.86:1, 1.91:1, and 2.16:1, respectively. The slight decrease in the metal ratio for the LDH–BT_CP might be attributed to the reaction conditions (pH = 9.5), which leads to the dissolution of the bentonite layers and prevents the formation of LDH nanoparticles, due to the presence of various dissolved metal ions from the bentonite [54,55]. In the case of the LDH–BT_ER, the LDH frameworks were slightly dissolved during the exfoliation–reassembly reaction in aqueous medium [56]. Notably, the metal ratio of LDH in the LDH–BT_SP was closely comparable to that of the parent LDH, thereby indicating that the LDH structure is well preserved after hybridization.

**Figure 2:**
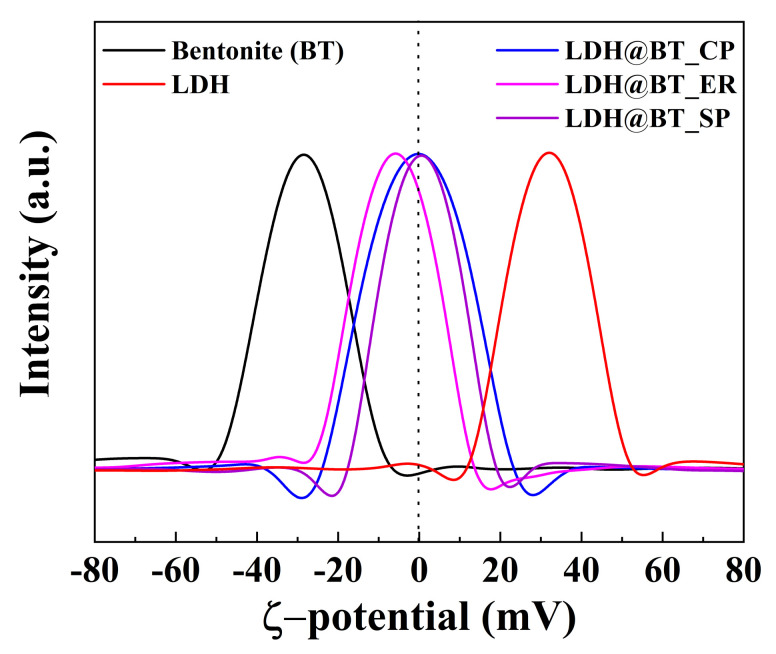
The zeta-potential distributions of the bentonite, LDH, LDH–BT_CP, LDH–BT_ER, and LDH–BT_SP. (dotted line indicating 0 mV).

The positive surface charge of LDH is a characteristic property that leads to the adsorption of anionic species, including oxyanions, via charge–charge interaction [57]. The zeta-potential distributions at pH 7.0 (Figure 2) indicate that the surface charges of the parent LDH and bentonite are +32.0 and −28.6 mV at pH 7.0. After hybridization, however, the average surface charges of the nanocomposites were shifted in a positive direction with respect to that of bentonite, with values of −0.29, −5.97, and +0.56 mV for the LDH–BT_CP, _ER, and _SP, respectively. This charge shift could be attributed to the presence of the LDH nanoparticles in the nanocomposites, and is expected to enhance the chromate adsorption efficacy of the bentonite.

**Figure 3:**
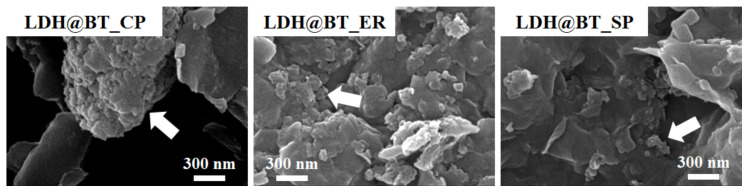
The SEM images of the LDH–BT_CP, LDH–BT_ER, and LDH–BT_SP (the white arrows indicate the LDH nanoparticles).

The morphologies of the pristine bentonite and LDH were revealed by the high-resolution SEM images in Supplementary Figure S2. Here, the bentonite exhibits typical few-μm aggregates with a plate-like morphology, while the LDH exhibits the conventional layered morphology with tens and hundreds of nm-sized platelets [58]. By comparison, the LDH–BT_CP (left-hand panel, Figure 3) exhibits a few μm-sized LDH particles that are inhomogeneously aggregated with the bentonite layers, whereas the LDH–BT_ER and LDH–BT_SP samples (middle and right-hand panels, Figure 3) exhibit homogenous distributions of LDH with bentonite after hybridization. It is notable that the simple solid-phase hybridization method led to a more homogeneous mixture of bentonite and LDH than the co-precipitation method did. Taken together, the PXRD, zeta-potential, and SEM results suggest that solid-phase hybridization could be a suitable eco-friendly methodology for the hybridization reaction of clay minerals, without the need for water or solvent, while preserving the crystal structure and morphology of each clay, along with a homogeneous distribution.

### 3.2. Chromate Adsorption Experiments

**Figure 4:**
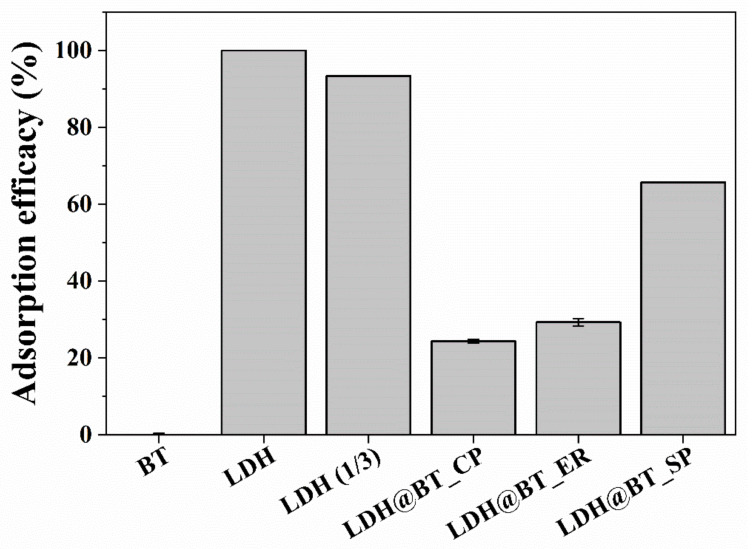
The chromate adsorption efficacies of the bentonite, LDH, LDH–BT_CP, LDH–BT_ER, and LDH–BT_SP (initial chromate concentration = 10 mg/L; adsorbent concentration = 1 g/L for the bentonite, LDH, LDH–BT_CP, _ER, and _SP, and 0.333 g/L for the LDH(1/3); contract time = 2 h).

The chromate adsorption efficacies of the pristine bentonite and LDH, as well as of the prepared nanocomposites, are presented in Figure 4. Here, the bentonite shows almost 0% chromate adsorption efficacy, which might be attributed to charge repulsion between the negatively charged bentonite and chromate ions. By contrast, the positively charged LDH nanoparticles exhibited 100% chromate removal efficacy, also probably due to adsorption via charge–charge interaction. Notably, when the dosing amount of LDH was decreased, almost the same contents of nanocomposites (≈33.3 wt.%) still showed around 93% of adsorption efficacy, indicating high chromate removal efficacy [59]. After hybridization, the LDH–BT_CP and LDH–BT_ER showed around 25–30% chromate adsorption efficacies, which might be attributed to a positive shift in the surface charge of the nanocomposite relative to that of bentonite due to the incorporation of the LDH nanoparticles. This is confirmed in Figure 4, where the LDH–BT_ER exhibits a 5% higher chromate adsorption efficacy than that of the LDH–BT_CP, due to the moderately homogeneous distribution of the LDH nanoparticles, while the LDH–BT_SP exhibits an exceptionally high chromate adsorption efficacy of around 65%. This is 2.6 times that of the LDH–BT_CP and 2.2 times that of the LDH–BT_ER, even though these two composites have similar surface charges; hence, it is also attributed to the homogeneous distribution of LDH. Based on the SEM images and zeta-potential analysis, the chromate adsorption efficacy on the LDH–BT nanocomposites was affected by the homogenous distribution of incorporated LDH particles and their surface charge.

**Figure 5:**
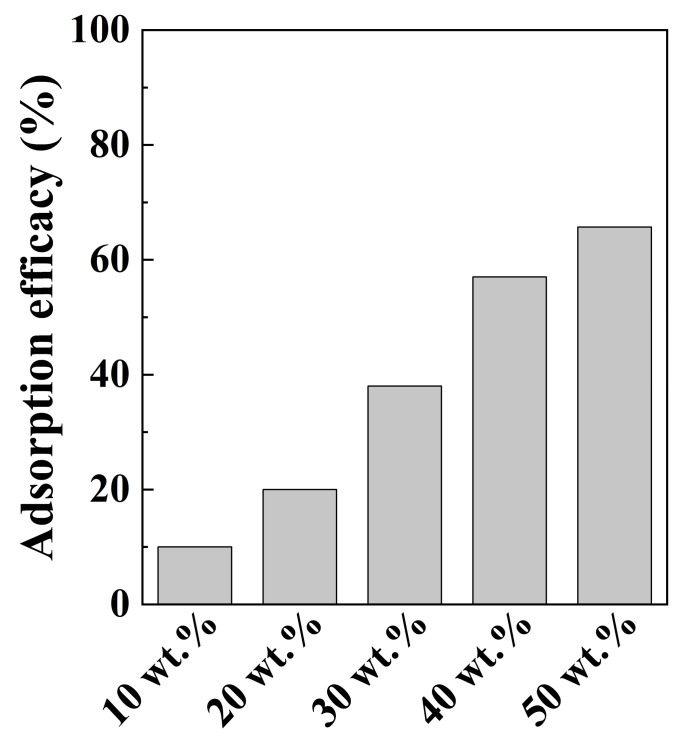
The chromate adsorption efficacies of LDH–BT_SP with various amounts (wt.%) of LDH (initial chromate concentration = 10 mg/L; adsorbent concentration = 1 g/L; contract time = 2 h).

To optimize the amount of LDH for solid-phase hybridization, the amount of added LDH was varied between 10 and 50% by weight of bentonite. The results in Figure 5 reveal a gradual increase in the chromate adsorption efficacy, from 10 to 65%. However, the increase in adsorption efficacy seems saturated at around 50 wt.% LDH (65% efficacy). These results demonstrate that the amount of LDH is a critical factor for effective chromate adsorption, and that 50% LDH by weight of bentonite could be the optimal condition for solid-phase hybridization.

**Figure 6:**
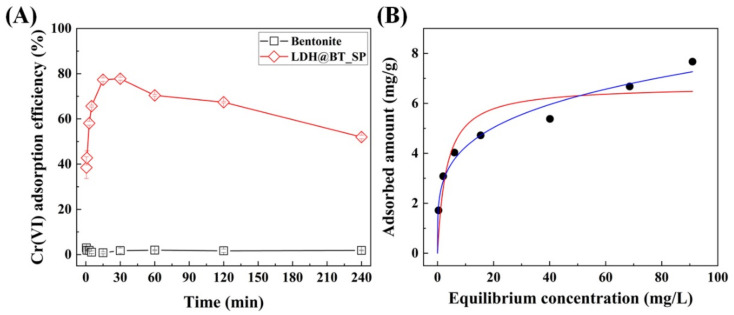
(**A**) The chromate adsorption efficiencies of the pristine bentonite and the LDH–BT_SP with 50 wt.% LDH (**A**) as a function of time (initial chromate concentration = 10 mg/L; adsorbent concentration = 1 g/L; contact time = 0.5–240 min). (**B**) The chromate adsorption isotherm results (black dot) and fitting profile of the LDH–BT_SP with the Langmuir (red line) and Freundlich (blue line) isotherm models (initial chromate concentration = 2–100 mg/L; adsorbent concentration = 1 g/L; contact time = 2 h).

The comparative chromate adsorption performances of the pristine bentonite and the LDH–BT_SP with 50 wt.% LDH were further evaluated via batch adsorption experiments with various contact times, and the results are shown in Figure 6A. Here, the pristine bentonite provides only around 1% chromate adsorption during 240 min, and this is consistent with the results in the previous batch test. By contrast, the LDH–BT_SP exhibits a rapid chromate adsorption efficacy of ~80% of fast chromate adsorption in the early stage (within 30 min, which then decreases gradually to ~52% after 240 min. These results can be explained by the diffusion of chromate ions from the bulk of the solution to the adsorbent surfaces. Due to the subsequent adsorption of these chromate ions by the LDH nanoparticles in the LDH–BT_SP, the active sites on the LDH could become saturated during the early stages, with some of the weakly bound chromate ions becoming desorbed during the later stages [60]. Nevertheless, the LDH–BT_SP with 50 wt.% LDH maintains a chromate adsorption efficacy of over 60%.

The chromate adsorption behavior of the LDH–BT_SP was evaluated by chromate adsorption isotherm experiments, depending on the concentrations of chromate solution, and the obtained results were analyzed by using the Langmuir and Freundlich isotherm models (Figure 6B). As summarized in Table 2, the calculated *q_m_* value from the Langmuir isotherm model was 6.705 mg/g, and the separation factor (*R_L_*) was 0.331, with the latter indicating that chromate adsorption by the LDH–BT_SP is a favorable reaction. The *q_m_* values of the reported clay-based adsorbents are summarized in Table S1. The obtained *q_m_* value of LDH–BT_SP was relatively higher than other natural clay-based adsorbents, indicating the advantage of hybridization between bentonite and LDH. The correlation coefficient (*R^2^*) values obtained from the Langmuir and Freundlich isotherm models are 0.8271 and 0.9742, respectively, thereby indicating that the chromate adsorption by LDH–BT_SP is more closely modeled by the Freundlich isotherm than by the Langmuir isotherm model. The results were interpreted with a Freundlich isotherm equation to evaluate the heterogeneous adsorption systems. From the Freundlich isotherm model, the calculated n value of 4.121 also indicates the favorability of the adsorption reaction on the LDH–BT_SP nanocomposite. According to these results, the LDH–BT_SP with 50 wt.% LDH exhibits multilayer adsorption by the heterogeneous surface, with a homogenous distribution of LDH nanoparticles on the surface of the bentonite [61].

### 3.3. Mobility Test

**Figure 7:**
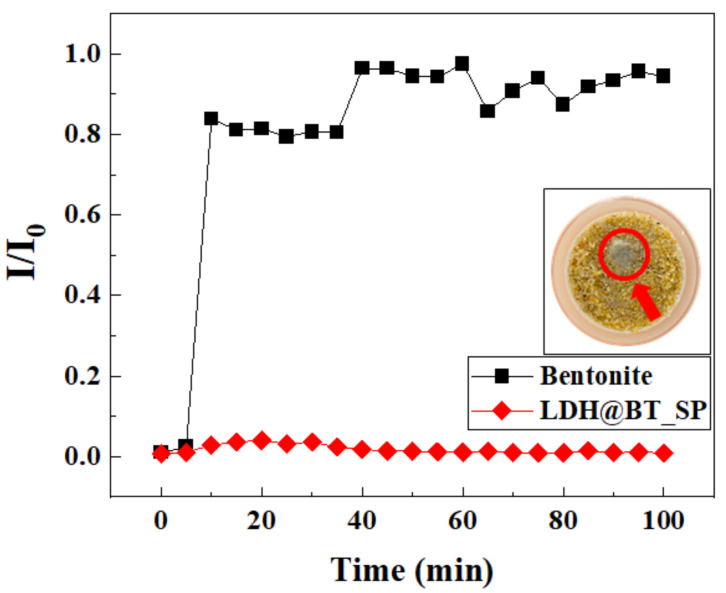
Mobility test in silica sand-filled column results of the pristine bentonite and the LDH–BT_SP under simulated soil conditions (adsorbent concentration = 1 g/L; flow rate = 5.0 mL/min). The inset is a photographic image of the top of the column 100 min after injection with the LDH–BT_SP, where the red arrow and circle indicate the LDH–BT_SP.

The mobility of a nanomaterial is an important factor for its utilization as a chromate adsorbent for in situ soil or groundwater remediation, as toxicity or unexpected side effects could become an issue if such nanomaterials in the soil can enter the food chain or drinking-water sources [62]. The mobilities of the pristine bentonite and the LDH–BT_SP were compared in Figure 7. Here, the pristine bentonite was released at around 10 min, and most of the bentonite particles were eluted during 100 min, thereby indicating the high mobility of bentonite in the silica-sand-filled column. The SEM image in Supplementary Figure S2 reveals that the pristine bentonite forms aggregates of around 5–10 μm, which are more than 100 times larger than the pristine LDH particles. However, the results herein show that the pristine bentonite is easily released from the silica sand-filled column, possibly due to the negative surface charges on both the bentonite and silica sand [63]. By contrast, almost no LDH–BT_SP is released from the column, thereby indicating that the composite particles remain entrapped in the pore structure of the silica sand. This might be attributed to interactions between the negatively charged silica sand and the positively charged LDH–BT_SP nanocomposites. These results suggest that the hybridization of bentonite with LDH might be effective at limiting the mobility of the nanocomposites in soil.

### 3.4. Chromate Adsorption under Simulated Sub-Surface Conditions

The chromate adsorption efficacy of the LDH–BT_SP under simulated subsurface conditions was carried out with a silica sand-filled box tester, as described in Supplementary Figure S1. In the absence of an adsorbent (control), the chromate ions were released immediately; their concentration increased to around 2.0 mg/L by 20 min and then decreased dramatically at 130 min, i.e., approximately 20 min after finishing the chromate solution injection. The total amount of released chromate ions was calculated to be around 280 mg/L·min (based on the area under the curve), which is close to the total amount of chromate solution input during 170 min (2.5 mg/L × 111.1 min ≈ 278 mg/L·min). This result indicates that the added chromate ions flow freely through the box tester without being adsorbed on the surface of sand filler.

**Figure 8:**
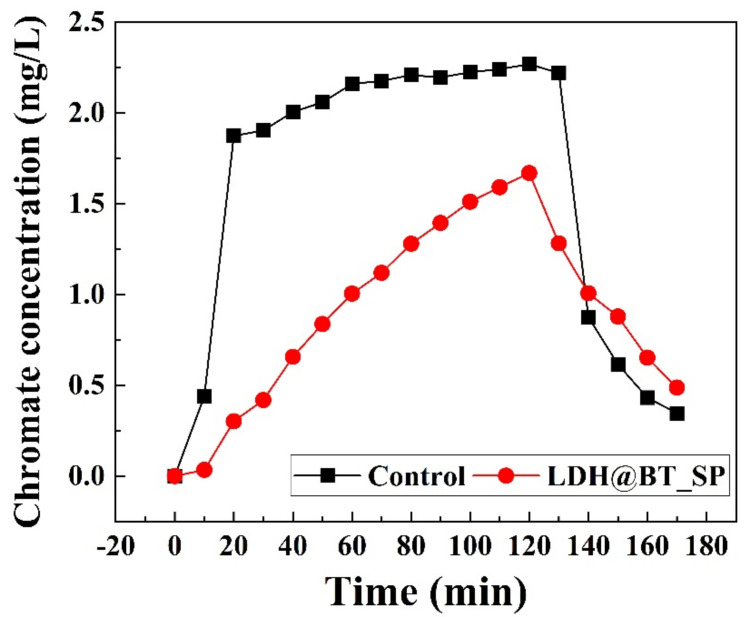
The time variation in chromate concentration in a silica-sand-filled box in the absence and presence of the LDH–BT_SP (adsorbent concentration = 10 g/L; flow rate = 9.0 mL/min).

When the LDH–BT_SP was injected into the middle of the box tester, it was expected to create a zone with LDH–BT_SP, acting as adsorbent for chromate ion decreasing the amount of chromate release. This is confirmed by the red profile in Figure 8, where the initial release of chromate was suppressed for 10 min, and the concentration then increased gradually from 0.30 mg/L at 20 min to 1.6 mg/L by 120 min. After 130 min, however, the chromate concentration began to decrease, reaching around 0.48 mg/L at 170 min. Based on the calculated area under the curve, the LDH–BT_SP released 158.8 mg/L·min, thereby indicating a 43.4% decrease in chromate release relative to the control experiment. These results further demonstrate that the composite of bentonite with LDH provides a potential chromate adsorbent for in situ soil and groundwater remediation.

## 4. Conclusions

Herein, nanocomposites of LDH and bentonite (LDH–BT) were successfully prepared via three different hybridization methods, namely co-precipitation (CP), exfoliation–reassembly (ER), and solid-phase (SP) hybridization. Preliminary characterization of the as-prepared LDH–BT_CP, _ER, and _SP via PXRD; SEM; and zeta-potentiometry revealed that the LDH–BT_SP retained the crystalline structures of the parent bentonite and LDH, that the LDH particles were homogeneously distributed on the bentonite, and that the surface of the composite was more positively charged than that of the pristine bentonite.

The chromate adsorption efficacy of the LDH–BT_SP was found to be 2.6 times that of the LDH–BT_CP and 2.2 times that of the LDH–BT _ER, representing an approximately 65% increase. From the study of chromate adsorption efficacy with various amounts of LDH in the LDH–BT_SP, the composition with 50 wt.% LDH was chosen for further investigation. While the pristine bentonite showed 0% chromate removal efficiency, the as-prepared LDH–BT_SP exhibited a fast chromate adsorption of 80% within 30 min. However, this was slightly desorbed during the subsequent 240 min, possibly due to weak binding of the chromate ions to the surface. Moreover, the fitting of the chromate adsorption profile by the Langmuir and Freundlich models demonstrated that the LDH–BT_SP follows the Freundlich isotherm, thereby indicating multilayer adsorption by the bentonite on the heterogeneous surface of the nanocomposites.

The mobility test in a silica-sand-filled column demonstrated that the high mobility of bentonite (around 100%) was dramatically decreased to ~1%, due to the positively charged surface of the LDH–BT_SP. Moreover, a chromate adsorption experiment under simulated subsurface soil conditions, using a silica-sand-filled box tester, demonstrated an approximately 43% efficiency during 170 min. Based on these results, the LDH–BT_SP could be an effective material for in situ soil and groundwater remediation. Furthermore, the solid-phase hybridization technique without the need for water or solvent is a potential method for the hybridization of two different clay materials in an eco-friendly way.

## Figures and Tables

**Table 1:** Detailed characterization results from the PXRD and elemental analysis.

Sample	d-Spacing (nm) (BT) ^a^	FWHM (degrees) (BT) ^a^	d-Spacing (nm) (LDH) ^b^	FWHM (degrees) (LDH) ^b^	Intensity Ratio ^c^	Mg:Al Ratio ^d^
Bentonite	1.24	2.378	–	–	–	–
LDH	–	–	0.78	1.583	–	2.13:1
LDH–BT_CP	1.45	0.617	0.78	1.642	4.73	1.86:1 *
LDH–BT_ER	1.27	0.905	0.78	1.524	10.25	1.91:1 *
LDH–BT_SP	0.99	0.980	0.78	1.502	1.75	2.16:1 *

^a^ Calculated from the (001) diffraction peak of bentonite. ^b^ Calculated from the (003) diffraction peak of LDH. ^c^ Obtained from the ratio of the (001) diffraction peak of bentonite to the (003) diffraction peak of LDH. ^d^ Calculated from the ICP–OES results. * Calculated based on the ICP–OES results excluding the Mg^2+^ and Al^3+^ from bentonite.

**Table 2:** The detailed parameters obtained from the Langmuir and Freundlich adsorption isotherm models.

Sample	Langmuir				Freundlich		
	*q_m_* (mg/g)	*a_L_* (L/mg)	*R_L_*	*R^2^*	*K_F_*	*n*	*R^2^*
LDH–BT_SP	6.705	3.191	0.331	0.8279	2.432	4.121	0.9742

## Data Availability

The data presented in this study are available on request from the corresponding author.

## Supplementary Materials

The following are available online at https://www.mdpi.com/article/10.3390/nano12081384/s1, Figure S1: Schematic diagram of chromate adsorption in simulated sub-groundwater condition in box tester, Figure S2: Scanning electron microscopy image of pristine bentonite and LDH, Table S1: Chromate adsorption capacities of clay-based adsorbents.
