# Supplementary material for: Adsorption of Chromate Ions by Layered Double Hydroxide–Bentonite Nanocomposite for Groundwater Remediation
Source: Nanomaterials (Basel). 2022 Apr 18;12(8):1384. doi: 10.3390/nano12081384 (PMC9025721; doi:10.3390/nano12081384)
Supplement: Supplementary file 1 [file nanomaterials-12-01384-s001.zip › nanomaterials-1681220-supplementary.pdf]

# Adsorption of Chromate Ions by Layered Double Hydroxide–Bentonite Nanocomposite for Groundwater Remediation

**Yoogyeong Kim [1], Yeongkyun Son [1], Sungjun Bae [2], Tae-Hyun Kim [1,*] and Yuhoon Hwang [1,*]**

[1] Department of Environmental Engineering, Seoul National University of Science and Technology, Seoul 01811, Korea; keo0610@seoultech.ac.kr (Y.K.); clayton7167@naver.com (Y.S.)
[2] Department of Civil and Environmental Engineering, Konkuk University, Seoul 05029, Korea; bsj1003@konkuk.ac.kr
* Correspondence: th.kim@seoultech.ac.kr (T.-H.K.); yhhwang@seoultech.ac.kr (Y.H.); Tel.: +82-2-970-6929 (T.-H.K.); +82-2-970-6626 (Y.H.); Fax: +82-2-971-5776 (T.-H.K. & Y.H.)

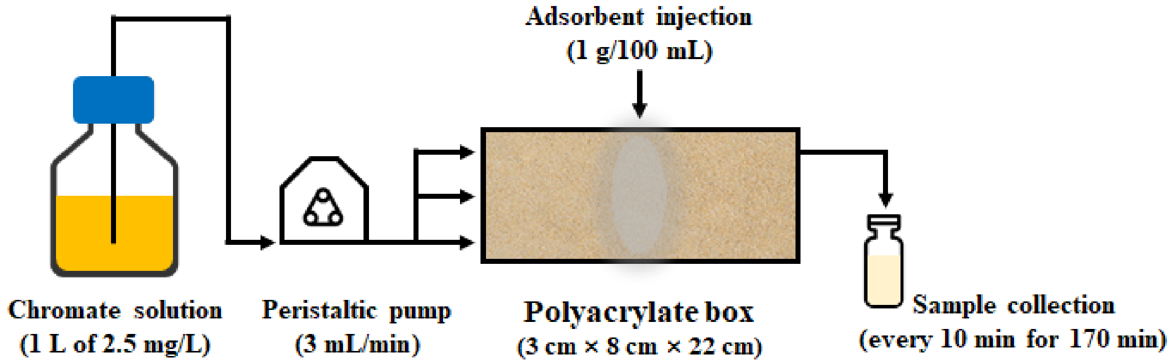


**Figure S1.** Schematic diagram of chromate adsorption in simulated sub-groundwater condition in box tester.

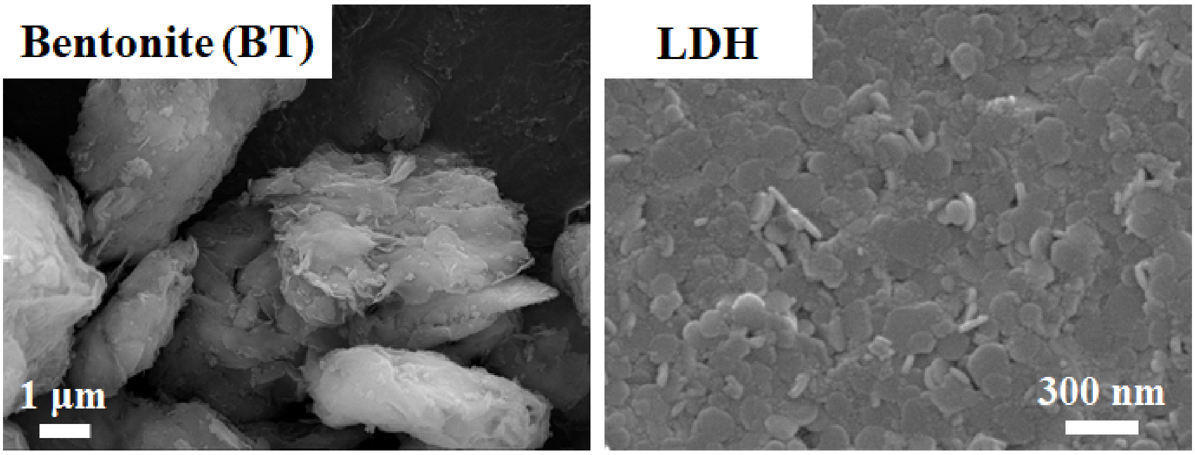


**Figure S2.** Scanning electron microscopy image of pristine bentonite and LDH.

**Table S1.** Chromate adsorption capacities of clay-based adsorbents.

| Adsorbents | $Q_m$[a] (mg/g) | Chromate Concentration (mg/L) | Contact Time (min) | Dosing (g/L) | References |
|---|---|---|---|---|---|
| Akadama clay | 4.29 | 50 | 180 | 5 | [1] |
| OTMAC-Bentonite | 12.40 | 40 | 60 | 6 | [2] |
| Shanghai silty clay | 1.85 | 10-250 | 1440 | 40 | [3] |
| Natural clay | 4.56 | 10-80 | 90 | 5 | [4] |
| Kaolinite | 0.571 | 0.1-16 | 1440 | 4 | [5] |
| Illite | 0.276 | 0.1-16 | 1440 | 4 | [5] |
| MgAl-Cl (LDH) | 58.82 | 5-200 | 120 | 2 | [6] |
| LDH@BT_SP | 6.705 | 2-100 | 120 | 1 | This study |

[a]Obtained from Langmuir isotherm fitting result.

## References

1. Zhao, Y.; Yang, S.; Ding, D.; Chen, J.; Yang, Y.; Lei, Z.; Feng, C.; Zhang, Z. Effective adsorption of Cr (VI) from aqueous solution using natural Akadama clay. *Journal of Colloid and Interface Science* **2013**, *395*, 198-204.

2. Zhang, S.; Yang, J.; Xin, X.; Yan, L.; Wei, Q.; Du, B. Adsorptive removal of Cr(VI) from aqueous solution onto different kinds of modified bentonites. *Environmental Progress & Sustainable Energy* **2015**, *34*, 39-46.

3. Wang, J.; Zhang, W. Evaluating the adsorption of Shanghai silty clay to Cd(II), Pb(II), As(V), and Cr(VI): kinetic, equilibrium, and thermodynamic studies. *Environmental Monitoring and Assessment* **2021**, *193*, 131.

4. Ashour, E.A.; Tony, M.A. Eco-friendly removal of hexavalent chromium from aqueous solution using natural clay mineral: activation and modification effects. *SN Applied Sciences* **2020**, *2*, 2042.

5. Ajouyed, O.; Hurel, C.; Marmier, N. Evaluation of the adsorption of hexavalent chromium on kaolinite and illite. *Journal of Environmental Protection* **2011**, *2*, 1347.

6. Khitous, M.; Salem, Z.; Halliche, D. Effect of interlayer anions on chromium removal using Mg–Al layered double hydroxides: Kinetic, equilibrium and thermodynamic studies. *Chinese Journal of Chemical Engineering* **2016**, *24*, 433-445.
